# Spinal Cord Injury AIS Predictions Using Machine Learning

**DOI:** 10.1523/ENEURO.0149-22.2022

**Published:** 2023-01-03

**Authors:** Dhruv Kapoor, Clark Xu

**Affiliations:** 1 College of Computing, Georgia Institute of Technology, Atlanta, Georgia 30332; 2 Department of Medicine, School of Medicine and Public Health, University of Wisconsin–Madison, Madison, Wisconsin 53705

**Keywords:** machine learning, NSCISC, prediction, recovery, spinal cord injury

## Abstract

The study used machine learning to predict The American Spinal Injury Association Impairment Scale (AIS) scores for newly injured spinal cord injury patients at hospital discharge time from hospital admission data. Additionally, machine learning was used to analyze the best model for feature importance to validate the criticality of the AIS score and highlight relevant demographic details. The data used for training machine learning models was from the National Spinal Cord Injury Statistical Center (NSCISC) database of U.S. spinal cord injury patient details. Eighteen real features were used from 417 provided features, which mapped to 53 machine learning features after processing. Eight models were tuned on the dataset to predict AIS scores, and Shapely analysis was performed to extract the most important of the 53 features. Patients within the NSCISC database who sustained injuries were between 1972 and 2016 after data cleaning (*n* = 20,790). Outcomes were test set multiclass accuracy and aggregated Shapely score magnitudes. Ridge Classifier was the best performer with 73.6% test set accuracy. AIS scores and neurologic category at the time of admission were the best predictors of recovery. Demographically, features were less important, but age, sex, marital status, and race stood out. AIS scores on admission are highly predictive of patient outcomes when combined with patient demographic data. Promising results in terms of predicting recovery were seen, and Shapely analysis allowed for the machine learning model to be probed as a whole, giving insight into overall feature trends.

## Visual Abstract

**Figure:**
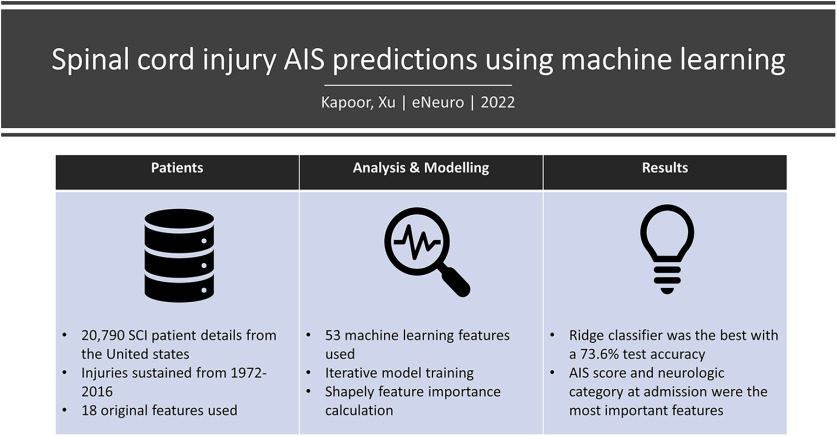

## Significance Statement

This research is intended to introduce the use of machine learning to enhance predictive capabilities of spinal cord injury recovery, to validate previous motor-sensory classification work, and to extract important deciders of recovery from constructed models.

## Introduction

Spinal cord injury (SCI) profoundly changes a patient’s life. Effects range from impaired motor function, up to and including paralysis of the limbs, as well as mental health effects such as depression or suicide. Patient outcomes are highly sensitive to where and to what extent the injury is on the spinal cord. In general, an injury closer to the brainstem has a greater impact. Among the impaired motor functions are the following: paralysis, loss of sensation, increased chance of developing pressure ulcers, bladder dysfunction, neurogenic bowel, muscle atrophy, autonomic dysreflexia, and impaired sexual function (Sezer et al., 2015).

The American Spinal Injury Association Impairment Scale (AIS) classifies the motor-sensory abilities of a patient with an SCI (Ho et al., 2007). There are five letter-grade categories, as follows: AIS grade A is a complete injury with no retention of motor control or sensory function below the point of injury; and AIS grade E is an injury with minimal impact on the patient. Clinicians use the AIS to classify SCI and quantify SCI recovery, for example, an improvement from grade B to C or a deterioration from grade D to C.

One key question among SCI patients is how the severity of their injury, as measured by the AIS, will improve or deteriorate during the course of SCI recovery. This varies by the type of injury and demographic differences between patients.

In a traditional clinical setting, the main factors identified for prognostication of SCI recovery include patient age, patient gender, length of inpatient stay, type of inpatient discharge, type of SCI, time to procedure, procedure type, and comorbidities (Chay and Kirshblum, 2020). SCI prognosis is primarily conducted by either standard of care diagnostics, including heuristic bedside evaluation and magnetic resonance imaging (MRI), or traditional clinical analysis, such as an odds ratio statistic (Burns et al., 2012). Thus, there is an opportunity to support SCI recovery by adding a machine learning-based framework of SCI prognosis using big data and precision medicine as one of the clinician’s tools for improving SCI patient outlook.

Researchers have developed many tools on SCI treatment; however, after literature review, it was seen that there is a wide gap in the use of machine learning algorithms to predict SCI recovery in a contemporary precision medicine context, especially with regard to feature importance and using a very large dataset (Snoek et al., 2004; Munce et al., 2014). One study attempted to predict discharge location using an ensemble model and used area under the curve as an outcome (Fan et al., 2021), another study made use of convolutional neural nets (CNNs) on MRI charts to achieve an accuracy of 71.4% (Okimatsu et al., 2022), while two other studies greatly limited the complexity to specific AIS scores of A (Buri et al., 2022) and D/E (Inoue et al., 2020). The authors in the study by Chou et al. (2022) conducted a study similar to the one presented here, but their sample size consisted of 74 patients.

The research carried forward was based on the National Spinal Cord Injury Statistical Center (NSCISC) database, which includes details from patients across the United States (Chen et al., 2016). Several different machine learning models were used to predict AIS level on patient discharge for data recorded between 1972 and 2016, and the best model was further examined to extract feature importance information. The ground truth AIS scores at discharge were supplied as part of the dataset.

The analysis of feature importance serves two purposes. One is to verify the importance of AIS classification at the time of hospital admission as a critical feature, using a data-driven approach. The other purpose is to identify demographic features that also play a crucial role in determining recovery.

## Materials and Methods

Computational implementation was conducted using Python version 3.8, shap version 0.40.0, and scikit-learn version 1.0.1.

### Data preparation

The NSCISC database comprises >29,000 traumatic SCIs since 1973 for patients treated at any regional model SCI system within the first year of injury and who have signed a consent form for inclusion (DeVivo et al., 2002). The patient details within the database have also been stripped of all identifiers defined by the HIPAA (Health Insurance Portability and Accountability Act of 1996). The NSCISC dataset was loaded from the published CSV format, and it had 417 raw features. A custom data mapper was used to translate the raw data headers and values into more recognizable features. For example, the label AWghtRhb was translated to “Weight at Admission.” Any nonrecorded values in the dataset were assigned a value of “Unknown.”

There were far more features in the dataset than are relevant to the machine learning models designed, so the data used for training was limited to patient information that is known at or before hospital admission. Exploratory data analysis showed that some features had >90% missingness, and these were excluded from consideration as model inputs. The other reasons for excluding certain variables within the dataset was whether they were specific to a certain area of the body or spine such as the sensory level of the left side during hospital admission. Including all of these would have greatly increased the sparsity of the eventual feature vector, leading to risks involved with the curse of dimensionality. Univariate analysis was also performed on possible features to look at maximum/minimum values, counts, and outliers. Table 1 shows the final features chosen as well as their mapped versions for input into machine learning model construction, along with imputations performed for missing values.

**Table 1:** Feature mappings from those in the original dataset to transformed versions used at model training time, along with the imputation techniques used

Original feature	Machine learning features	Missing value imputation technique
Occupation status—injury	Homemaker, in training, in workshop, other, retired, student or infant, unemployed, unknown, working	Mapped to unknown
Diabetes—history	Same as original feature	Mode (no history)
Veteran	Same as original feature	Mode (not a veteran)
Race	Asian, black, multiracial, Native American, unknown, white	Mapped to unknown
AIS—admission	A, B, C, D	None; rows dropped
Sex	Same as original feature	None; all values were populated
Education—injury	Same as original feature	Mode (high school)
Depression—history	Same as original feature	Mode (no history)
TBI likelihood—injury	Same as original feature	Mode (improbable)
Level of injury—admission	Same as original feature	None; rows dropped
Daily alcohol—history	Same as original feature	Mode (zero)
Anxiety—history	False, general anxiety, multiple, PTSD, panic disorder, unknown	Mapped to unknown
Primary insurance	True, false, unknown	Mapped to unknown
Age—injury	Same as original feature	None; all values were populated
Loss of memory—injury	Same as original feature	Mode (no loss of memory)
Marital status—injury	Divorced, living unmarried with partner, married, never married, other, separated, unknown, widowed	Mapped to unknown
Neurologic category— admission	Complete paraplegic, complete tetraplegic, incomplete paraplegic, incomplete tetraplegic, minimal deficit paraplegic, minimal deficit tetraplegic, unknown	Mapped to unknown
Loss of consciousness— injury	Same as original feature	Mode (no loss of consciousness)

TBI, Traumatic brain injury.

Generally, imputation followed the format of using the mode as the chosen mapped value or an “Unknown” label was assigned instead if it had already been found in the set of values of a feature. The choice to use mode over creating a new Unknown label when not already available was decided because of two primary reasons. The first reason was to avoid creating a value that very few rows have, which could have the side effect of incorrectly flagging these patients as disproportionately important during training time. The second was to avoid inflating feature dimensionality for features such as education level at injury because the introduction of an Unknown value would require a transition from an ordinal feature to a one-hot encoded feature. Sex and age at injury were the only features that were fully populated, whereas AIS score and level of injury at hospital admission were the only features where a missing value resulted in a dropped row.

From analysis, the typical profile of an SCI patient was found to be a male, between 19 and 29 years of age, white, and never married, although the dataset showed plenty of variation from this modal profile.

Of the dataset features, three variables were determined as the most suitable for measuring SCI recovery through the patient’s course of treatment, as follows: AIS score, neurologic disposition at hospital discharge, and patient’s level of injury. These three features were suitable because they could capture patient physical improvements throughout the entire body. The AIS score at discharge was ultimately chosen because of its widespread use in the literature (Roberts et al., 2017; Inoue et al., 2020; Buri et al., 2022; Chou et al., 2022; Okimatsu et al., 2022). Using this target variable meant that a five-class classification modeling approach was to be designed, where each class is one of A, B, C, D, or E. With this definition, there was a possibility that some patient predictions could include worsening of the AIS score as well. In the dataset, only 329 of these cases were found in total, 275 in the eventual training set and 54 in the eventual testing set, for a combined ∼1.6% of all samples. As a result, the vast majority of predictions was focused on recovery.

The finalized dataset after analysis that was used for model training is described in Table 2. Four hundred seventeen raw NSCISC features were reduced to 18, and later mapped to 53 model-ready features. The train/test split of ∼90:10 was reached after trialing different ratios and evaluating test accuracy.

**Table 2:** Final dataset at a glance that was used for machine learning

Description	Value
Total samples	20,790
Training samples	18,737
Training injury dates	1972–2005
Testing samples	2053
Testing injury dates	2006–2016
Original features	18
Machine learning features	53

### Modeling and feature importance

**Figure 1.**
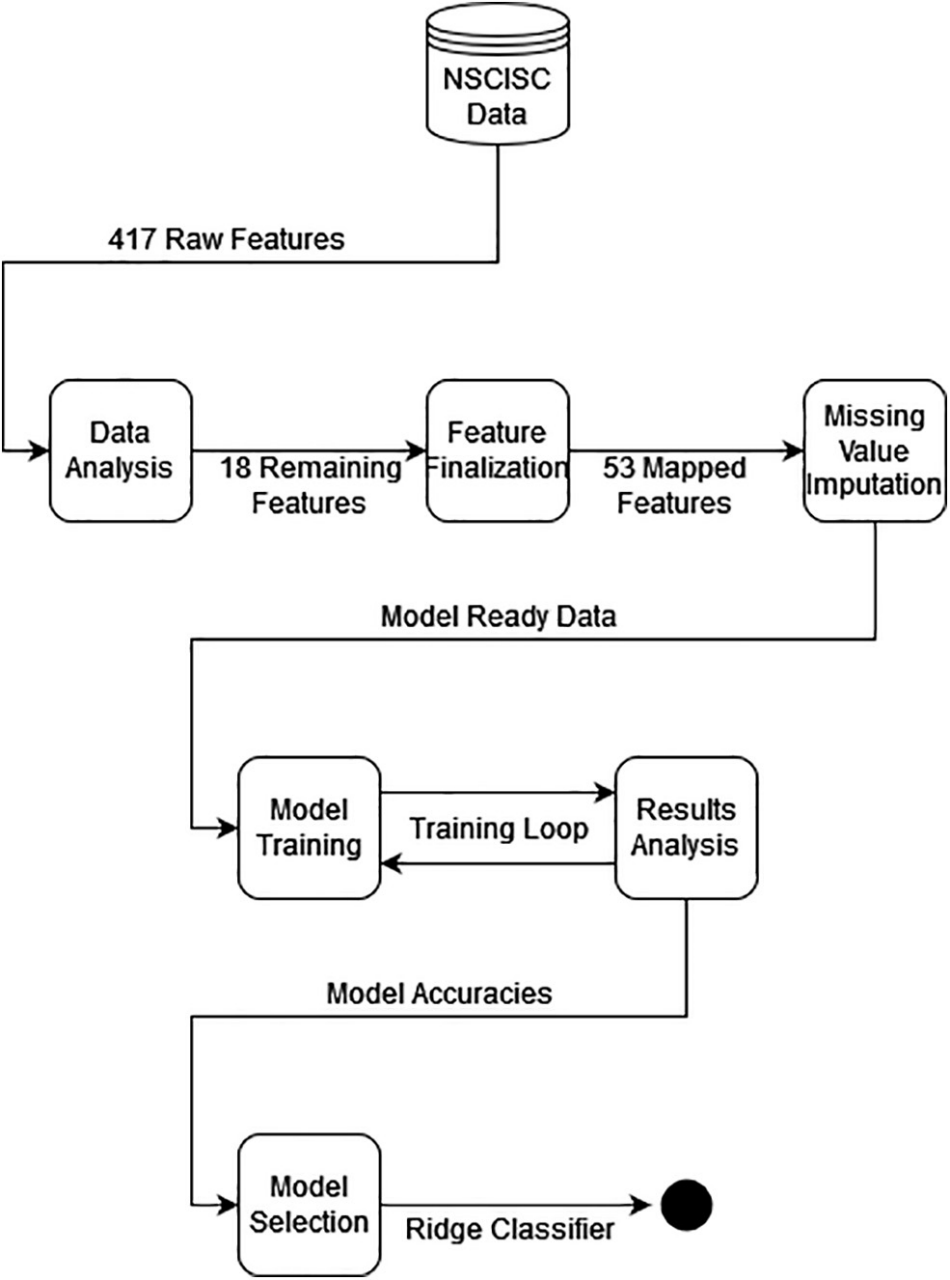
Data preparation flowchart through to model selection (full image is available at: https://github.com/kapoor1992/spinal_cord_injury_recovery/blob/release/submission/src/ml/modelling/plots/flowchart.png).

There were eight different machine learning models tuned after the dataset was prepared, and test set prediction accuracy was the decider in determining the best model. The process of data preparation through to model selection is outlined in Figure 1.

To extract feature importance from the top model, Shapely values were used (Parsa et al., 2020). These provide a quantitative measurement of how strong a certain feature is in predicting a specific class output. The magnitude of the Shapely value was used so feature strength for or against a specific class is captured. These values were computed per sample and taking the mean over all samples can provide an average importance. The strength of each of the 53 features with respect to a given class is thus given as follows:

(1) $$\forall f,c:S_{f,c}=\frac{\sum_{i=0}^{N}|{\text{shap}}_{f,c,i}|}{N},$$

where *f*, *c*, and *N* correspond to feature, class, and number of samples, respectively. Feature strength can be summed across all classes to give an overall importance metric with respect to the model as a whole, as seen in the following:

(2) $$\forall f:S_{f,m}=\overset{C}{\underset{i=0}{\sum }}S_{f,i},$$

where *m* and *C* are the model and the set of all classes, respectively.

## Results

The results after training the data on all tuned models are described in Table 3. With a multiclass test accuracy of 73.6%, it was found from the machine learning model metrics that the best performing model was Ridge Classifier over the NSCISC dataset for SCI recovery prognostication. SVM, Elastic Net, and Logistic Regression closely followed with 73.5%, 73.2%, and 73.2%, respectively. These results are very promising, given that they are only for information discovered or provided on an initial assessment. Taking the Ridge Classifier and applying it to the dataset once again, but with removing the 329 patients who had lower AIS scores at hospital discharge than at admission gave a higher multiclass test accuracy of 75.3%.

**Table 3:** Accuracy results from machine learning model runs, ordered in descending order by test accuracy

Model	Train accuracy	Test accuracy
Ridge Classifier	0.824	0.736
SVM	0.825	0.735
Elastic Net	0.823	0.732
Logistic regression	0.824	0.732
Ensemble (Elastic Net, KNN, Random Forest)	0.860	0.717
CNN	0.840	0.711
Random Forest	0.927	0.693
Naive Bayes	0.591	0.429

**Figure 2.**
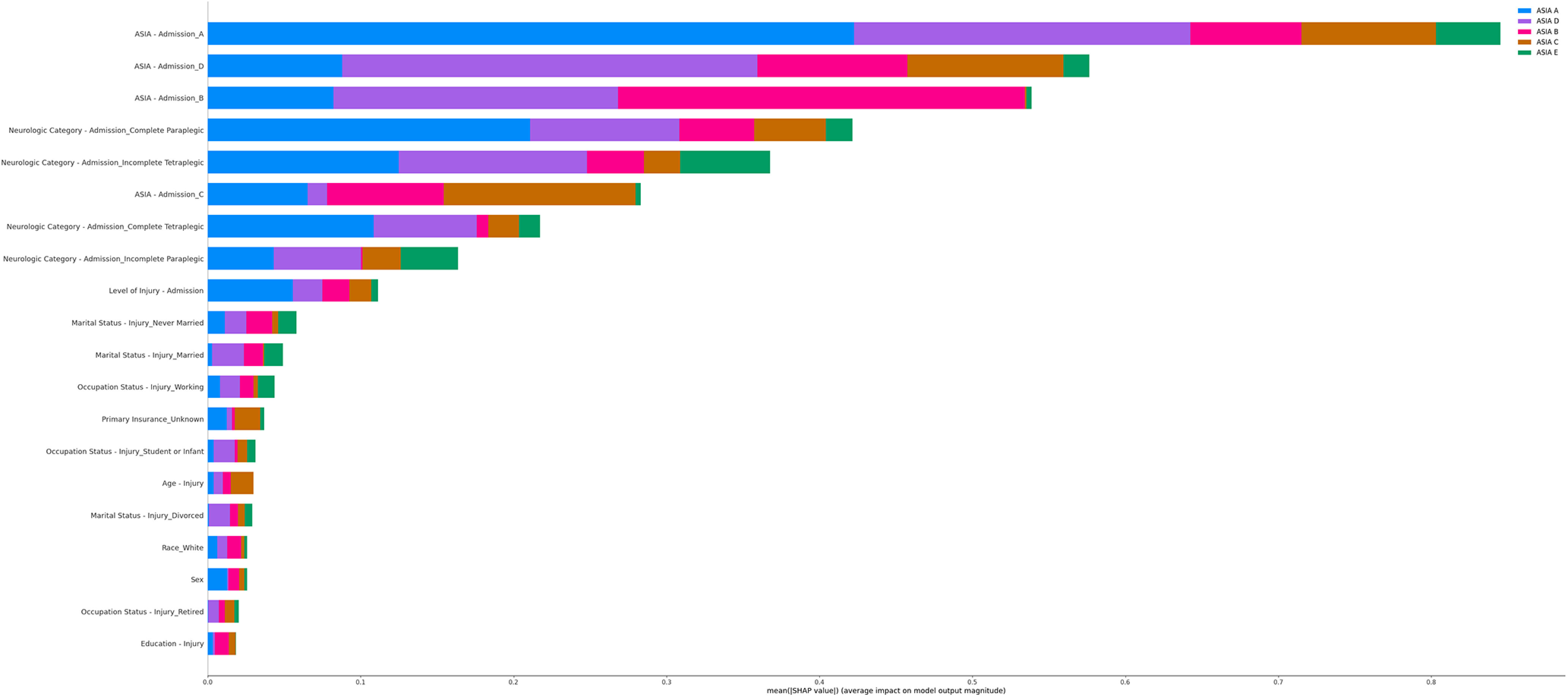
Top 20 most important features (full image is available at: https://github.com/kapoor1992/spinal_cord_injury_recovery/blob/release/submission/src/ml/modelling/plots/importance.png).

To determine what inputs are most crucial to predicting recovery, Shapely values were applied, as per Equation 1 and Equation 2, over the Ridge Classifier model. The top 20 most important features are visualized in Figure 2 and are recorded in Table 4. It has been previously shown that the best indicator of AIS score at hospital discharge is generally the AIS score at hospital admission and neurologic category at admission (Chay and Kirshblum, 2020), and the results confirm this.

**Table 4:** Top 20 most important features for Ridge Classifier

Rank	Machine learning feature
1	AIS—admission_A
2	AIS—admission_D
3	AIS—admission_B
4	Neurologic category—admission_complete paraplegic
5	Neurologic category—admission_incomplete tetraplegic
6	AIS—admission_C
7	Neurologic category—admission_complete tetraplegic
8	Neurologic category—admission_incomplete paraplegic
9	Level of Injury—admission
10	Marital status—injury_never married
11	Marital status—injury_married
12	Occupation status—injury_working
13	Primary insurance_unknown
14	Occupation status—injury_student or infant
15	Age—injury
16	Marital status—injury_divorced
17	Race_white
18	Sex
19	Occupation status—injury_retired
20	Education—injury

In terms of demographic details playing a role, these were less impactful. From previous research, it was expected that age would be among the most important (Seel et al., 2001; Wilson et al., 2012, 2014). While it was, there were a number of other features, such as sex and race, which were also around the same level of absolute mean importance. Interestingly, age was of a disproportionately higher importance for AIS grade C predictions. And, unexpectedly, marital status showed to be even more important than age.

## Discussion

The results showed promising results in predicting AIS improvement. A 73.6% test accuracy can be considered a benchmark for improvement. Given the high number of data samples, there is also the option of more complex models to trial, such as deep neural networks. The use of more engineered features can also be used as a tool to add more insight while reducing dimensionality. For example, height and weight features can instead be replaced with a body mass index measure. Finally, while the SCI recovery predictions is the result of one machine learning model in the pipeline, additional improvements in model performance could be gained by the creation of a model of models in which multiple submodels optimize for predicting features of importance to SCI recovery, which then feed into an overall model for prognosticating the patient.

One attempt made to increase performance was by dropping the 1.6% of patients whose conditions deteriorated in terms of AIS score. The boost ended up being 1.7% to test the accuracy to put it at 75.3%. This was likely because cases where patient scores deteriorated were difficult for the model to appropriately fit. There is the opportunity to use this as a secondary model if patients have shown signs of AIS recovery before hospital discharge. Otherwise, the original model is more appropriate since it makes no assumptions about progression. Nonetheless, the amount of increase in test accuracy exceeded expectations, considering that this difference between Ridge Classifier and the next three best performing models of this metric in the original dataset, was, at most, 0.4%.

Feature strength gave a better understanding of which areas most affected recovery, both when it came to validating the importance of the hospital admission AIS score and with regard to understanding the role that demographics play. The surprising result of marital status exceeding the importance of age was an important outcome of the study. The presence of a support system for a patient may be a critical component of recovery success, though this would need to be examined more closely in future work. However, an important note for the Shapely analysis is to remember that the values computed from real data on SCI recovery show that there may be socioeconomic factors at play that act as social determinants of health among disadvantaged and underserved groups. As a result, whether these demographic details are highlighted as strong or weak features may actually be partially or completely because of societal complexities.

Comparing the results with those found in the study by Okimatsu et al. (2022), the test accuracy here of 73.6% is an improvement over the MRI CNN accuracy of 71.4%. Furthermore, the Ridge Classifier is much easier to interpret and is a more time-efficient model to train. The inclusion of imagery from MRI as a set of features is a possible route of future research that could further bolster the Ridge Classifier as well. In contrast to the research performed in studies by Inoue et al. (2020), Fan et al. (2021), Buri et al. (2022), Chou et al. (2022), and Okimatsu et al. (2022) as a whole, the study conducted here uses a patient base one to two times larger, while including a comprehensive review of feature importance as well. To add on, the results are, overall, comparable or better while considering all AIS classes and using a very lightweight model that can be much more easily deployed.

To extend the machine learning research into SCI recovery outlined in this article, the codebase found at https://github.com/kapoor1992/spinal_cord_injury_recovery and also available as the Extended Data 1 can be augmented by including more models or altering input features before metrics are recomputed.

There are a few limitations with the dataset that can be a point of future research. First, the features did not look at information past hospital admission time. The inclusion of new variables during an inpatient stay may be important to evaluate how much these initial features vary in importance. For example, the amount of weekly physical therapy, the amount of counseling that patients receive, and others could drastically alter results. The scope of the current article only captured a snapshot of prognosis at initial intake and evaluation of the newly injured patient. Also, in terms of model performance and tuning as more data are extracted from the NSCISC dataset, there is a growing opportunity to measure accuracy or other metrics in a longitudinal study. This can help to identify the limitation that the underlying NSCISC data are a static data source, which does not have the capability for either batch or streaming updates to the data. Therefore, the quality of the machine learning model predictions may decay over time as the SCI patient population experiences underlying demographic shifts.

10.1523/ENEURO.0149-22.2022.ed1 Extended Data 1 Spinal cord injury recovery-release-submission. Download Extended Data 1, ZIP file

## Synthesis

Reviewing Editor: Mark Laubach, American University Decisions are customarily a result of the Reviewing Editor and the peer reviewers coming together and discussing their recommendations until a consensus is reached. When revisions are invited, a fact-based synthesis statement explaining their decision and outlining what is needed to prepare a revision will be listed below. The following reviewer(s) agreed to reveal their identity: Michael Fehlings.

Your manuscript was reviewed again by one of the previous reviewers. They recommended revisions. Please address all points that were raised. I will review your revised manuscript before making a final decision on its acceptability for publication. Thank you for sending your work to eNeuro.

Visual Abstract: Please consider providing a visual abstract. This helps attract readers to your paper.

Reviewer Comments

Advances the Field: Although there are limitations to the included data, such as the absence of variables including time to surgery and hospital transfer status, which have been shown to have an impact on AIS conversion, the study pulls from a large dataset to support machine learning. The result is a well performing model with good test set accuracy of over 70%. The authors have responded adequately to previous comments by reviewers.

Statistic: Statistical methods are valid for prediction. Sensitivity analysis recommended by excluding portion of dataset in which AIS declines.

Comments to the Authors:

- It is informative to know the imputation approach for each variable in the table. Please include the details of imputation approach in the manuscript.

- Please ensure, for proper flow of the manuscript, that interpretations of results are in the discussion section e.g. lines 149 to 153 and 160-162.

- I recommend adding a limitations subsection to the discussion. It seems like limitations are discussed sporadically through all sections. It would improve the organization of the paper to have them collated in one section.

- I recommend a sensitivity analysis in which you exclude the 1.3% of dataset with a decline in AIS scores and re-running your analysis to test the robustness of your findings.

## Author Response

We have carefully reviewed the comments left by the reviewers of our submission and have addressed the points they made. One by one, below is how we resolved each one.

1. Imputation approach required.

Table 1 was previously created, which shows how missing values were filled in for every feature. We have now also dedicated a paragraph in the Methods section explaining why mode or Unknown was used as well as recapping features that did not require imputation.

2. Interpretation of results should be moved to the Discussion.

The previous lines 149-152 and 160-162 have now shifted to the appropriate section.

3. Create Limitations under Discussion.

This subsection now sits at the end of Discussion and some previously mentioned limitations in the discussion section have moved there as well.

4. Rerun analysis after removing patients whose scores declined.

We ran our best model after removing the suggested patients and recorded the test accuracy under Results and also added commentary in Discussion. Note, we previously had 275 written as the total number of these types of patients but a slight correction was made because that was only in the training set. The code has also been updated to run this analysis.
