# Supplementary figures and images for: Spinal Cord Injury AIS Predictions Using Machine Learning
Source: eNeuro. 2023 Jan 3;10(1):ENEURO.0149-22.2022. doi: 10.1523/ENEURO.0149-22.2022 (PMC9831144; doi:10.1523/ENEURO.0149-22.2022)

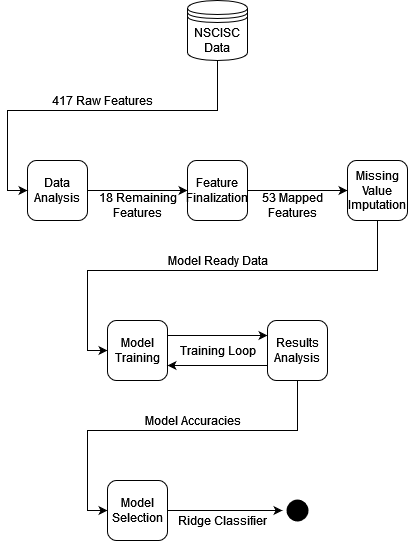

Supplement: Extended Data 1 — Spinal cord injury recovery-release-submission. Download Extended Data 1, ZIP file [file enu-eN-NWR-0149-22-s01.zip › spinal_cord_injury_recovery-release-submission/src/ml/modelling/plots/flowchart.png]

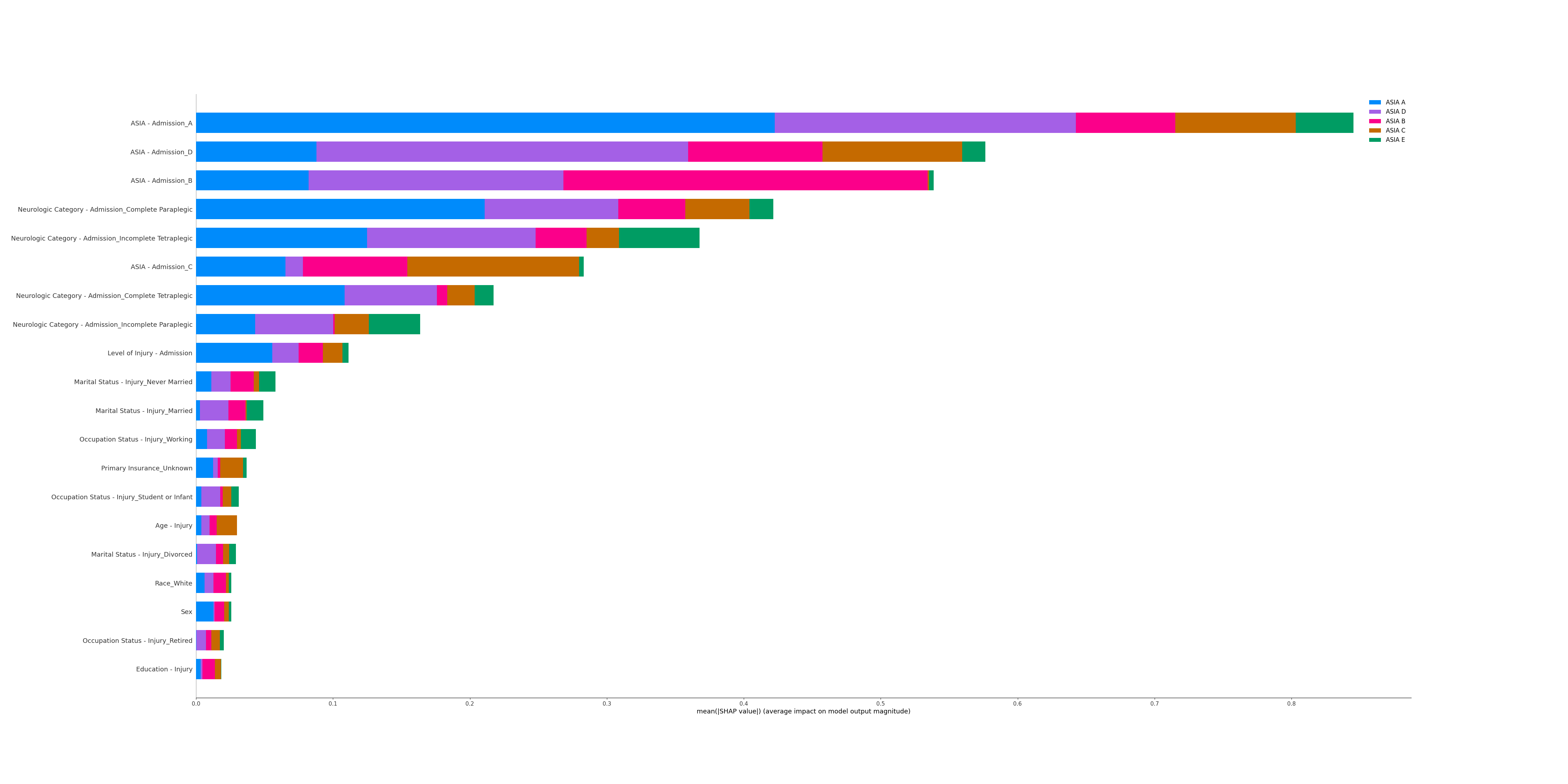

Supplement: Extended Data 1 — Spinal cord injury recovery-release-submission. Download Extended Data 1, ZIP file [file enu-eN-NWR-0149-22-s01.zip › spinal_cord_injury_recovery-release-submission/src/ml/modelling/plots/importance.png]

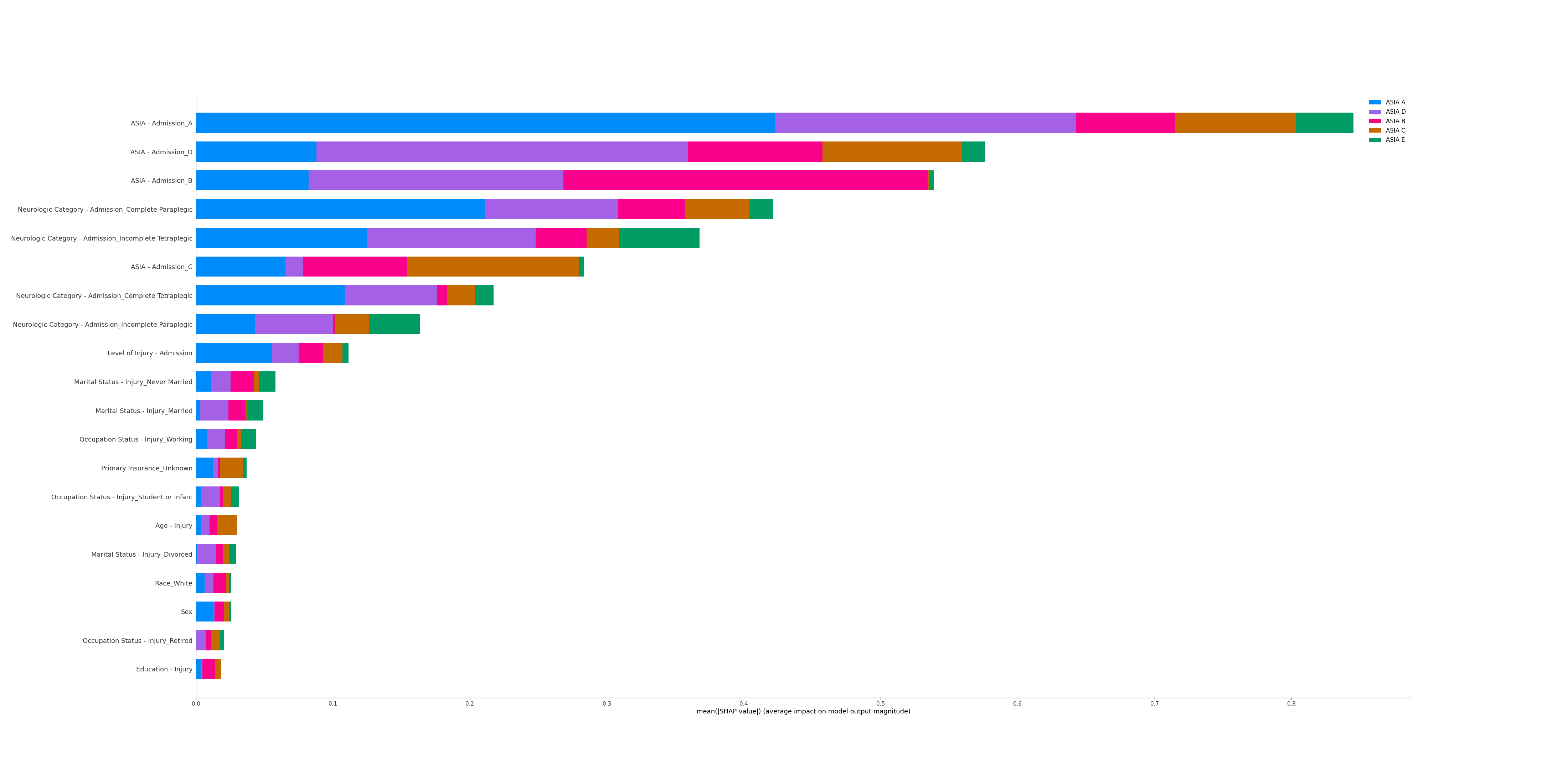

Supplement: Extended Data 1 — Spinal cord injury recovery-release-submission. Download Extended Data 1, ZIP file [file enu-eN-NWR-0149-22-s01.zip › spinal_cord_injury_recovery-release-submission/src/ml/modelling/plots/importance.tiff]
